# Acute Massive Gastrointestinal Bleeding Caused by Ascaris lumbricoides Infection: A Case Report

**DOI:** 10.7759/cureus.30935

**Published:** 2022-10-31

**Authors:** Munsef Barakat, Ahmed Shebani, Amin Rehman

**Affiliations:** 1 Internal Medicine Residency Program, Medical Education, Hamad Medical Corporation, Doha, QAT; 2 Internal Medicine, Hamad Medical Corporation, Doha, QAT

**Keywords:** obscure gastrointestinal bleeding, video capsule endoscopy, intestinal nematode, acute gastrointestinal bleeding, ascaris lumbricoides infection

## Abstract

*Ascaris lumbricoides* infestation can cause a variety of intestinal complications, but severe gastrointestinal bleeding is rare. A thorough evaluation is needed for travelers and migrants with massive gastrointestinal bleeding, especially in those patients who have undergone multiple upper and lower endoscopies with no certain cause. We present a challenging case of massive small bowel bleeding due to Ascaris infection.

## Introduction

Gastrointestinal bleeding is a fairly common presentation and has been traditionally categorized into upper and lower gastrointestinal bleeding in relation to the ligament of Treitz. Lower gastrointestinal bleeding occurs distal to the ligament of Treitz and is commonly caused by large bowel malignancy, colitis, diverticular disease, anorectal diseases, and angiodysplasia [1]. Small bowel bleeding, formerly known as obscure gastrointestinal bleeding, represents a minority of cases of gastrointestinal bleeding (5-10%) [2] and has remained a diagnostic challenge over the years prior to the development of advanced small bowel imaging.

*Ascaris (A.)* *lumbricoides* (roundworm) is a nematode that causes an infection transmitted mainly through the ingestion of contaminated water and food and is mainly seen in poor and developing countries with poor sanitation [3]. Infection with roundworm can cause a variety of symptoms, including respiratory symptoms, malabsorption, malnutrition, and anemia, which can affect children's growth [4]. Massive gastrointestinal bleeding is a rare manifestation, with only a few cases reported so far. We present a challenging case of Ascaris infection manifested by small bowel bleeding.

## Case presentation

A 40-year-old man from Southeast Asia presented to the emergency department with a three-day history of multiple large episodes of melena not associated with vomiting, nausea, or hematemesis and no hematochezia. No history of any chronic medication other than brief use of non-steroidal anti-inflammatory drugs (NSAIDs) for upper respiratory infection one week earlier was noted. The patient is a lifelong non-smoker and has never consumed alcohol.

At the time of examination, he was alert and not in distress. Pale conjunctiva was seen. His vitals were stable, well perfused with normal capillary refill time, with no tachycardia or orthostatic blood pressure drop, and there were no clinical signs of hypovolemic shock. The abdominal examination was unremarkable, and a digital rectal examination revealed melaena with no fresh blood. Otherwise, his exam was normal.

Laboratory investigations revealed that his hemoglobin level was 6.7 gram/dL with normal platelet and white cell counts with a normal coagulation profile. The patient was administered 2 units of packed RBCs and started on an intravenous proton pump inhibitor infusion (PPI). The next day, emergency upper and lower gastrointestinal endoscopies were performed and found no signs of active or recent bleeding.

**Figure 1:**
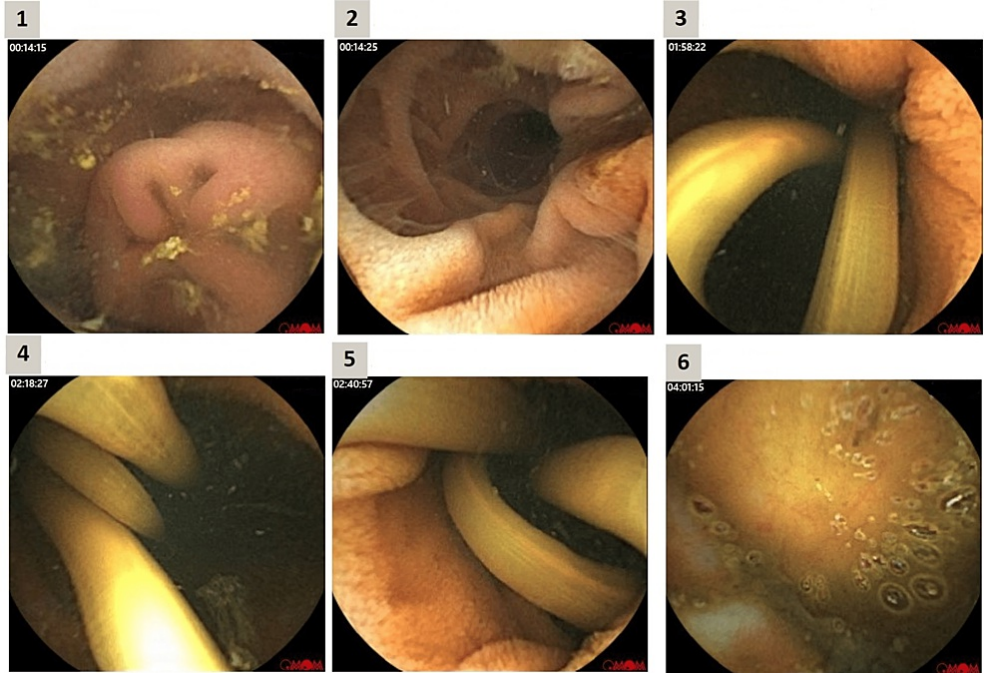
Video capsule endoscopy (VCE) showing intestinal Ascaris lumbricoides 1, Duodenum, 2, Ampulla of Vater. 3-5, Ascaris lumbricoides in the jejunum. 6, Cecum

By this time, a suspicion of small bowel bleeding had risen. Push enteroscopy was arranged the next day and examination up to the proximal jejunum was unremarkable. As the patient was stable with no further drop in hemoglobin after observation, arrangements were made for a video capsule endoscopy (VCE) as an outpatient (Figure 1).

A few days after discharge, the patient was readmitted to the ED with multiple episodes of new-onset hematochezia and melena. On examination, he was pale and sweaty with tachycardia and hypotension (blood pressure 85/60mmHg, HR = 120 bpm). Hemoglobin dropped to 6.6 gram/dL, from the previous 10 gram/dL, so rapid transfusion protocol was activated, and the patient received 6 units of Packed RBCs with platelets and fresh frozen plasma and was admitted to the intensive care unit and kept on PPI intravenous infusion.

An emergency computed tomography (CT) angiogram did not demonstrate any active bleeding. Repeated upper endoscopies and colonoscopies were normal. A capsule endoscopy was performed after vital stabilization, and it revealed multiple long roundworms in the small bowel (*A. lumbricoides*). The patient has been treated with a single dose of albendazole 400 mg and no further drop of hemoglobin was observed and was discharged.

During the follow-up appointment, the patient didn’t report any melena or hematochezia and his complete blood count remained normal.

## Discussion

*A. lumbricoides* is one of the most common causes of parasitic infections worldwide. The life cycle of the Ascaris species starts with the ingestion of the egg through fecal-oral contamination. After the eggs hatch in the organs, the larvae penetrate the intestinal wall to enter portal circulation and migrate through the liver all the way to the lungs. Later, the larvae migrate through the bronchi and are swallowed to enter the intestinal tract to mature into adult roundworms [5].

Ascaris causes a wide range of gastrointestinal symptoms, ranging from malabsorption and failure to thrive in children, intestinal obstruction with heavy intestinal manifestations, especially in young children, pancreatitis, obstructive jaundice, and cholangitis. Chronic intestinal infestation can cause chronic occult blood loss and iron deficiency anemia [6]. Acute gastrointestinal blood loss is rare and has been reported only in a few cases [3].

In our patient, he presented with melena with a drop in hemoglobin. Commonly, melena is caused by upper gastrointestinal bleeding as it takes time for blood to be digested in the GI tract. Given the presentation and the history of brief non-steroidal anti-inflammatory drug use, an upper endoscopy was arranged, which was observed to be normal with no evidence of recent bleeding. A colonoscopy was performed as a part of the evaluation, which was normal as well.

Small bowel bleeding is a relatively rare cause of gastrointestinal bleeding and requires a systematic approach when suspected. A second look at upper and lower endoscopy is needed along with push endoscopy. If the patient is clinically unstable, angiography should be performed immediately. if the patient is stable then video capsule endoscopy (VCE) is a good alternative, but when an intestinal obstruction is suspected. Computed tomographic enterography should be done first [2].

Our patient continued to exhibit overt gastrointestinal blood loss with a drop in hemoglobin. A CT angiogram was performed and presented normal findings. After stabilization, video capsule endoscopy revealed evidence of Ascaris infection, which was treated with a single dose of albendazole.

Although no clear bleeding or ulceration was seen on VCE examination, it was assumed that the cause of the blood loss was likely the mechanical trauma of the intestinal mucosa due to the worms’ attachment and the resultant abrasion.

## Conclusions

Ascariasis is a common infection worldwide with a variable gastrointestinal presentation, although it is commonly known to cause iron deficiency anemia due to chronic gastrointestinal blood loss, massive acute GI bleeding appears to be rare. In the right clinical and epidemiological context, ascariasis should be kept in the differential diagnosis.

## References

[1] DiGregorio AM, Alvey H (2022). Gastrointestinal Bleeding. https://www.ncbi.nlm.nih.gov/books/NBK537291/.

[2] Gerson LB, Fidler JL, Cave DR, Leighton JA (2015). ACG clinical guideline: diagnosis and management of small bowel bleeding. Am J Gastroenterol.

[3] Sangkhathat S, Patrapinyokul S, Wudhisuthimethawee P, Chedphaopan J, Mitamun W (2003). Massive gastrointestinal bleeding in infants with ascariasis. J Pediatr Surg.

[4] Hagel I, Giusti T (2010). Ascaris lumbricoides: an overview of therapeutic targets. Infect Disord Drug Targets.

[5] Dold C, Holland CV (2011). Ascaris and ascariasis. Microbes Infect.

[6] Walter BM, Born P, Winker J (2015). Ascaris lumbricoides causing obscure gastrointestinal bleeding detected by double-balloon enteroscopy. Endoscopy.

